# Temporal dynamics of N_2_O emissions from pasture urine patches: drivers and source processes

**DOI:** 10.1007/s10705-026-10498-w

**Published:** 2026-05-19

**Authors:** Lena Barczyk, Markus Jocher, Julius Havsteen, Joachim Mohn, Johan Six, Christof Ammann

**Affiliations:** 1 Agroscope Research Station, Climate and Agriculture Group, CH-8046 Zurich, Switzerland; 2 Laboratory for Air Pollution/Environmental Technology, Empa, CH-8600 Dübendorf, Zurich, Switzerland; 3 Department of Environmental Systems Science, ETH Zürich, CH-8092 Zurich, Switzerland

**Keywords:** Cattle urine patch, Nitrous oxide, Environmental driver, Source processes

## Abstract

Nitrous oxide fluxes from urine patches (F(N_2_O)_urine_) of grazing livestock are variable over time due to fluctuations in driving parameters, such as soil temperature, water-filled pore space (WFPS), and availability of the source substrates ammonium and nitrate. Therefore, the frequency and timing of flux measurements after urine application are important when determining cumulative F(N_2_O)_urine_. In this study, F(N_2_O)_urine_ was measured in eight experiments at high temporal frequency using an automatic chamber system in a pasture located in Switzerland. A driver analysis using random forest identified the time since urine application as most important predictor for F(N_2_O)_urine_. The exponential decay in F(N_2_O)_urine_ after urine addition was in parallel to decreasing soil ammonium but anticorrelated to nitrate concentration, suggesting that nitrification and nitrifier denitrification are major source processes. Since nitrate showed elevated concentrations up to 122 days after application, bacterial denitrification is most likely responsible for late F(N_2_O)_urine_ peaks following an increase of WFPS. The isotopic composition of the emitted N_2_O indicates that nitrification dominates the N_2_O production immediately after urine application, while bacterial denitrification and nitrifier denitrification become more important with increasing time since urine application. The observed high-frequency emission time series were also used to simulate typical low-frequent manual chamber sampling schedules. They led to considerable deviations (up to ± 30%) of the time-integrated N_2_O emission. However, the average bias was considerably smaller (− 5%) for the prescribed sampling schedule, indicating that manual chamber measurements can reasonably quantify average total N_2_O emissions from urine patches under the present pedoclimatic conditions.

## Introduction

Nitrous oxide (N_2_O) is an important greenhouse gas (GHG), substantially contributing to the anthropogenic-driven climate change (IPCC 2023). Intensively managed grasslands are important sources for N_2_O due to mineral N applications, manure N applications and livestock excreta depositions (Dangal et al. 2019). The deposition of urine by grazing livestock, in particular, involves highly localized input rates of nitrogen (N), reaching values of up to 2000 kg N ha^−1^ (Haynes and Williams 1993; Welten et al. 2013; Selbie et al. 2015). The N input by urine, is influenced by factors such as feed composition or lactation stage (Firkins and Reynolds 2005; Dijkstra et al. 2013). The N loading rates in urine patches are considerably higher than plant requirements. Therefore, urine N is prone to causing environmental problems via leaching or gaseous emissions as ammonia (NH_3_), nitric oxide (NO), nitrous oxide (N_2_O), or N loss via di-nitrogen (N_2_) (Whitehead and Raistrick 1993; Hubbard et al. 2004; Cai and Akiyama 2016).

Urea makes up the largest proportion of total urine N (Spek et al. 2012, 2013; Moraes et al. 2017) and converts into ammonium (NH_4_^+^) within hours to few days after application to the soil by reacting with water and the soil enzyme urease. This process, named hydrolysis, leads to a fast increase in soil pH (Reynolds et al. 1985; Cabrera et al. 1991; Bussink and Oenema 1998). Nitrous oxide can be emitted through both the biological oxidation of NH_4_^+^ via hydroxylamine (NH_2_OH), nitrite (NO_2_^−^) to nitrate (NO_3_^−^) (nitrification) and the reduction of NO_3_^−^ via NO_2_^−^ and N_2_O to N_2_ (bacterial denitrification). Nitrification and bacterial denitrification are considered as major pathways of N_2_O production in agricultural soils (Braker et al. 2011; Syakila and Kroeze et al. 2011; Butterbach-Bahl 2013). Other pathways of N_2_O formation like chemo-denitrification (Spott et al. 2011; Wei et al. 2019), fungal denitrification (Rohe et al. 2017) or nitrifier denitrification (Wrage-Mönnig et al. 2018) are less well studied but may lead to substantial N_2_O emissions under specific conditions. Microbial pathways are controlled by a number of factors, such as soil pH, available C, soil temperature, soil oxygen and moisture (Butterbach-Bahl 2013). Largest N_2_O emissions and emission peaks often occur after precipitation and at very high water-filled pore space values, which are associated with bacterial denitrification as major pathway (Dobbie et al. 1999; Luo et al. 2015). However, in urine patches, large NH_4_^+^ concentrations favor the oxidation of NH_4_^+^ to NO_2_^−^, while hypoxic conditions enhance the subsequent reduction of NO_2_^−^ to nitric oxide (NO), nitrous oxide (N_2_O) and molecular nitrogen (N_2_) through nitrifier denitrification (Wrage-Mönnig et al. 2018; Clough et al. 2020).

Advances in laser spectroscopic techniques have enabled measurement of singly substituted N_2_O isotopologues (^14^N^15^N^16^O, ^15^N^14^N^16^O, ^14^N^14^N^18^O) at ambient concentrations. These isotopic tracers provide important information for identification of various microbial N_2_O production pathways (Yu et al. 2020). Optical spectroscopy offers advantages with respect to site-selectivity (^14^N^15^N^16^O vs. ^15^N^14^N^16^O), ease of use and real-time analysis. However, obtaining high-quality N_2_O isotope data remains challenging, since the raw measurement data requires extensive post-processing due to a complex interplay arising from spectral interferences, gas composition, fitting algorithms and instrumental parameters (Harris et al. 2021). As a result, the uptake of N_2_O stable isotope techniques by research labs is still limited and hardly any such measurements exist for urine patches (Carter 2007).

At the national scale, total N_2_O emission estimates from urine patches are usually based on emission factors (EF) that quantify N_2_O emissions as the proportion of applied urine N in excess of background N_2_O emissions (IPCC 2006). IPCC suggests an average global default value for EF_urine_ of 0.77%, which is commonly used. But a number of countries use country- or region-specific values ranging from 0.06% in Western Canada to 1.2% in Ireland (Lemke et al. 2012; Krol et al. 2016; Chadwick et al. 2018; van der Weerden et al. 2020). EF values for urine patches (EF_urine_) are commonly based on manual flux chamber measurements. Specific N_2_O emissions from urine addition are determined by comparing emissions of treatments with urine application versus background fluxes on small spatial areas typically < 1 m^2^ (de Klein et al. 2020). Manual chamber systems can be used in a spatially flexible way, and thus are able to assess the spatial variability of N_2_O fluxes. In most studies, however, the sampling frequency of manual chambers is only fortnightly or weekly, and two to three times per week directly after N fertilization events, due to time and cost of labour (Chadwick et al. 2014; Krol et al. 2016; Charteris et al. 2020). Therefore, manual chambers might not be able to adequately capture the temporal dynamics of fluxes, including peak emissions. In contrast, automatic chamber techniques allow measurements with a much higher temporal resolution. However, they require higher technical effort and on-site online trace gas measurements and are therefore, usually more limited in the number of spatial repetitions (Laville et al. 2017; van der Weerden et al. 2013; Grace et al. 2020).

In the present study, we conducted field experiments with an automated chamber system measuring N_2_O emissions of a synthetic urine application event with a time resolution of about 4 h. The objective of the study was to investigate the detailed temporal dynamics of N_2_O emissions from urine patches and to relate them to relevant drivers. The latter included the evolution of soil NH_4_^+^ and NO_3_^−^ contents in urine patches, which were monitored at the same site but with a lower time resolution (weekly to monthly). In one selected experiment, the isotopic composition of the emitted N_2_O was measured to identify major microbial production pathways. We further used the high-frequency emission time series to evaluate whether typical temporal sampling schedules used for manual chamber measurements can capture N_2_O flux dynamics and cumulative emissions of urine patches in a satisfactory way.

## Methods

### Study site

The study was conducted between 2020 and 2024 on a grazed pasture of 2.8 ha located in North-Eastern Switzerland (47°29′26.7″N, 8°55′12.1″E; 517 m elevation) that has been already described in detail by Barczyk et al. (2023, 2024). The climate is temperate with an annual average temperature of 9.5 °C and an annual average precipitation of 1124 mm (2009–2019) (MeteoSwiss 2022). The site has been a permanent pasture system since 2013. It is rotationally grazed from April to October by Brown Swiss and Red Holstein dairy cattle with occasional grass cuts. For each measurement year, a different area of approximately 0.13 ha was excluded from grazing to perform urine application experiments. Measurements started at least 6 months after the last grazing activity of the previous year to minimize the influence of old excreta patches.

### Urine application experiments

Controlled applications of synthetic urine were performed separately for the following three purposes: (1) experiments UAC1–UAC8 for continuous N_2_O flux measurements by automated chambers between 2021 and 2023, (2) experiments U1–U10 for about weekly sampling of soil mineral N (N_min_) between 2020 and 2022, and (3) experiment UIso for N_2_O isotopic composition measurements in 2024 (see details in following sections; Table 4). The applications for the three purposes were mostly not synchronous due to the following reasons: The N_min_ sampling was mainly performed in connection to manual chamber measurements (Barczyk et al. 2023) starting in 2020, while the automated chamber system to measure continuous N_2_O fluxes was only available from August 2021 onwards at the site, and the instrument for analysing the isotopic composition of N_2_O gas samples was available from 2024 onwards. Therefore, the results can be related to each other only in an aggregated form.

The same synthetic urine composition and application rate were used for all experiments. The urine was produced one day prior to application and was stored at 4 °C overnight. The standard urine solution contained 10 g of N (91% as urea and 9% as hippuric acid), as well as 14 g KHCO_3_, 10.5 g KCl, 0.4 g CaCl_2_*2H_2_O, 1.2 g MgCl*5H_2_O and 3.7 g Na_2_SO_4_ [L^−1^] in order to mimic real urine properties (Kool et al. 2006). Urine was applied in 2 L within circular patch areas of 0.12 m^2^ in mineral N experiments, while it was applied in 1.5 L within rectangular chamber areas of 0.09 m^2^. The application rate was 167 g N/m^2^, corresponding to the standard application rate in Barczyk et al. (2023). Thus all application experiments performed in this study are well comparable concerning the soil type and general pasture conditions as well as the urine patch characteristics. Yet, with the different application times during the grazing seasons of the study period they represent a range of different soil temperature and moisture conditions.

### Automated chamber flux measurements

An automated non-steady-state flow-through chamber system based on a previous version described by Flechard et al. (2005) and Ammann et al. (2020) was used to measure N_2_O fluxes continuously from urine patches and from untreated control areas in eight experiments conducted between August 2021 and February 2023 (see Table 4). The stainless-steel chambers of 300 mm × 300 mm × 250 mm size were placed on PVC frames inserted 50 mm into the soil. The insertion of chamber frames into the soil occurred at least two weeks prior to urine application. Flexible silicone at the bottom and a foam band at the top sealed the chambers to prevent leakage of headspace air.

The alternating closing and opening of the chamber lids were controlled by a customized LabVIEW (National Instruments, US) program. The lid of an individual chamber closed every 4 h for 10 min. During chamber closure, air from the chamber headspace was continuously circulated through two 20 m polyamide tubes to the measurement container and back to the chamber using a bypass pump (flow rate 1.5 L min^−1^). Parallel to the bypass pump, a fraction (0.2 L min^−1^) of the circulation flow was directed through a gas concentration analyzer (G2308, Picarro, USA). The dry air N_2_O (as well as CO_2_ and CH_4_) concentrations were recorded at a time resolution of 10 s by the LabView program.

N_2_O fluxes F(N_2_O) were calculated over five minutes (minute 2–6) of chamber closure time with the HMR package in R (Pedersen et al. 2010) using linear and nonlinear regression similarly as described in Barczyk et al. (2023). The effective minimal detectable flux (MDF) was statistically estimated according to Barczyk et al. (2023), using all positive fluxes that were not significantly different from zero based on their 95% confidence intervals (CI). 95% of flux subsample data points fell below 2.8 μg N_2_O-N m^−2^ h^−1^, which was thus assigned as MDF. For fluxes below the MDF, always the linear regression result was selected, while for fluxes above the MDF either the linear or nonlinear result was used based on the automated model selection of the HMR package. Fluxes were rejected if ratios of F(N_2_O)_nonlinear_/F(N_2_O)_linear_ were > 4 (Hüppi et al. 2018) or if the coefficient of determination (R^2^) of the corresponding CO_2_ flux was < 0.8. In addition, N_2_O fluxes were rejected if both the relative uncertainty was ≥ 25% and the absolute uncertainty > MDF.

The urine application treatments and untreated control areas were both measured with 3–4 replicate chambers arranged in a spatially alternating or randomized way (corresponding to a randomized block design). Continuous and synchronous time series for the flux of the urine application treatment (F(N_2_O)_treatment_) and of the untreated control areas (F(N_2_O)_control_) were determined by averaging over the replicate chambers using a running average with a window length of two hours or extended to include at least six individual flux measurements. Then the urine related flux F(N_2_O)_urine_ was calculated as the difference between the running averages of F(N_2_O)_treatment_ and F(N_2_O)_control_. Finally, the cumulative urine related emission flux ∑F(N_2_O)_urine_ was determined by integration over time. The uncertainty of the cumulative emission results was estimated from the variability of the cumulative emissions (calculated by linear interpolation) of the individual replicate chambers.

### Flux driver analysis by random forest

A non-parametric random forest (RF) machine-learning algorithm was used to investigate the effect of drivers on the temporal evolution of F(N_2_O)_urine_ with a half-hourly time resolution. The following variables were tested as predictors: time since urine application t_appl_ [days], soil temperature T_s_ [°C], soil water-filled pore space WFPS [%], and precipitation P [mm] cumulated over the last 2, 6, 12, 24, 48 h before N_2_O measurement. The precipitation options were chosen as a proxy for soil moisture increases close to the surface, which were not fully represented by the corresponding measurements. Soil volumetric water content (VWC) and T_s_ in 50 mm depth were measured continuously at the experimental site by GS3 (Decagon Devices Inc.) and ML3 (Delta-T Devices Ltd.) probes. WFPS was calculated using total pore volume determined from soil samples taken at the site. Furthermore, a weather station at the site recorded precipitation.

The RF algorithm was run via *train* function (method *rf*) in the R package *caret* with ten-fold cross-validation in ten repetitions, and by using a training dataset containing 75% of data. The remaining 25% of data was used as a test dataset for validation. The number of randomly selected predictors at each node in the tree (*mtry*) was optimal for a value of 3. RF models were trained by various predictor combinations and various time intervals for predictor P. The final model was selected based on the predictive ability using coefficient of determination (R^2^), root mean squared error (RSME) and mean absolute error (MAE). Feature importance was assessed via *varImp* function in the *caret* package. To analyse the dependence of F(N_2_O)_urine_ on predictor variables, partial dependence plots were produced (R package *pdp*).

### Soil mineral nitrogen sampling

Soil samples of urine treatments and untreated control plots for N_min_ analysis were taken in eight urine application experiments between autumn 2020 and spring 2022 with a minimum of three repetitions per treatment. Two liters of standard synthetic urine were uniformly applied to circular areas of 0.12 m^2^. Urine application dates of experiments U1-U10 are listed in Table 4. The naming of the experiments (U1–U10) was adopted from Barczyk et al. (2023), although U2 and U8 did not include soil sampling and thus were not included here. Barczyk et al. (2023) also provides information on environmental conditions during the experiments.

The sampling of soil N_min_ in 0–50 mm depth took place at regular time intervals (weekly to biweekly) after urine application starting 1–4 days after urine application by using a Puerckhauer auger with a diameter of 18 mm. The fresh soil was prepared for soluble N_min_ extraction by removing stones and coarse roots. 20 g of the fine soil was extracted with 80 ml 0.01 mol L^−1^ CaCl_2_ solution. The NO_3_^−^-N and NH_4_^+^-N concentrations in the extractions were measured photometrically by SAN^++^ flow injection analyzer (Skalar Inc.) according to international standardizations ISO 11732:2005 and ISO 13395:1997. Dry soil NO_3_^−^-N and NH_4_^+^-N contents were calculated by using the volumetric water content measured by ML3 probes (Delta-T Devices Ltd.) in 0–50 mm depth at the experimental site. For each experiment, average control NO_3_^−^-N and NH_4_^+^-N contents were subtracted from the respective urine treatment values to get the urine related soil mineral N contents.

### Analysis of N_2_O isotopologues

With the purpose to identify the relevant microbial N_2_O production processes, gas fluxes and N_2_O isotopic signatures were determined in one experiment with urine application conducted on 08 August 2024. Three static chambers with the same design as described above for the online automated flux measurements were used. Yet, they were not connected to an online gas analyzer but operated as automatic time integrating chamber (ATIC) systems accumulating chamber air sampling in gas bags according to Wang et al. (2022; 2025). In short, after each chamber closure, the ATIC system automatically collects sequential gas samples into impermeable 5 L gas bags (30,228-U, Supel™-Inert Multi-Layer Foil, Sigma-Aldrich, USA). For the presented study the following timings were applied; each chamber was closed for 15 min every 4 h; during each closure event, four consecutive headspace samples were collected for 15 s, with sampling for Bag_1_ beginning at 3.50 min after closure, followed by filling of the subsequent bags (Bag_2_, Bag_3,_ Bag_4_) at 7.25, 11.50 and 14.25 min, respectively. Sample air in the four bags was accumulated over multiple chamber closures during typically one week. Then the sample bags were exchanged and brought to the lab for analysis.

Gas concentrations (N_2_O, CO_2_) and singly substituted isotopic composition of N_2_O (δ^15^N^α^, δ^15^N^β^, δ^18^O) were analyzed using commercial analyzers (G2401-m for CO_2_ and G5131-*i* for N_2_O, δ^15^N^α^, δ^15^N^β^, δ^18^O; Picarro Inc., USA). The raw data were corrected for spectral interferences, instrumental drift, and calibrated against established scales provided by WMO GAW for greenhouse gas concentrations and Air-N2 (^15^N/^14^N) as well as VSMOW (^18^O/^16^O) for N_2_O isotopologues (Mohn et al. 2022). Details on the correction and calibration approach is provided in Havsteen et al. (2025). Analysis of δ^18^O is also provided by the G5131-*i* instrument but did not show sufficient accuracy and therefore was not interpreted further in the present study.

This analytical workflow enabled the calculation of weekly mean fluxes of N_2_O and CO_2_ per chamber using a linear regression approach for gas concentration over time, with an imposed quality criterion of R^2^ ≥ 0.7 for data acceptance. The concentration-to-flux conversion considered dynamic molar volumes, calculated from the mean ambient temperature over the measurement period, recorded at a weather station located at the same field site.

The microbial N_2_O isotopic source signatures were derived using a two-endmember mixing model (Keeling plot), where each gas sample collected from a chamber represents a mixture of ambient atmospheric N_2_O and N_2_O from soil-derived microbial production. To determine the microbial source endmember for a specific time chamber, isotopologue values were plotted against the reciprocal N_2_O concentrations of the respective bags. The intercept of the regression line provides an estimate of the microbial N_2_O source signature, i.e. δ^15^N^α^ (^14^N^15^N^16^O) and δ^15^N^β^ (^15^N^14^N^16^O) from which δ^15^N^bulk^ = (δ^15^N^α^ + δ^15^N^β^)/2 as well as δ^15^N^SP^ = (δ^15^N^α^-δ^15^N^β^) were calculated (Yu et al. 2020). To assess the data quality, the regression-derived isotopic composition at ambient N_2_O concentration was compared to actual values. Three quality classes were defined, class 1 for agreement within 4 ‰, classes 2 and 3 within 6 ‰ and 8 ‰. Regression lines with deviations in ambient isotopic signatures from actual values of more than 8 ‰ were excluded from further analysis. To avoid false positive results under low flux scenarios, an additional criterion was applied, requiring a minimum N_2_O flux threshold of 75 µg m^−2^ h^−1^ (much higher than the MDF presented above).

### Simulating the sampling regime of manual chamber measurements

The continuous high-frequency N_2_O flux measurements by the online automated chambers were used to simulate the sampling time schedule of manual chambers. For this purpose, urine application experiments with measurement lengths > 30 d were selected to cover the main part of the total cumulated emissions ∑F(N_2_O). In the first scenario (Sim1), F(N_2_O)_urine_ data points were selected according to the sampling regime described in Barczyk et al. (2023) and linearly interpolated to calculate cumulative emissions ∑F(N_2_O)_urine_sim_. Barczyk et al. (2023) performed manual chamber measurements on urine patches 1–2 h past urine application followed by one-day and two-day intervals in the first week and afterwards by weekly and finally fortnightly measuring intervals (i.e. measurements at t_appl_ = {0, 1, 2, 4, 6, 8, 15, 22, 29, 36, 50, 64, …}d).

In the second scenario (Sim2), measurement intervals were kept as in Sim1, but with a more flexible timing to specifically sample after significant rainfall events with more than 10 mm d^−1^ to capture emission peaks. In this case, samples are taken one day after the precipitation event.

In the third scenario (Sim3), measurement intervals were kept as in Sim2, but it was sampled again three days after the rainfall events to grasp the decline of emission peaks as recommended by van der Weerden et al. (2013) and, therefore, to diminish possible overestimation of cumulative fluxes in Sim2.

To estimate daily average N_2_O fluxes, manual chamber measurements are recommended to be carried out between 10 am and 12 pm (Charteris et al. 2020). Therefore, ∑F(N_2_O)_urine_sim_ of Sim1 and Sim2 and Sim3 were determined as an average of samplings at 10 am, 11 am and 12 pm. Since it is not recommended to do manual chamber measurements under heavy rainfall, the day of sampling was shifted to the next day if the cumulative rainfall was > 2 mm between 10 am and 12 pm.

## Results

### Continuous N_2_O fluxes and environmental conditions

**Fig. 1:**
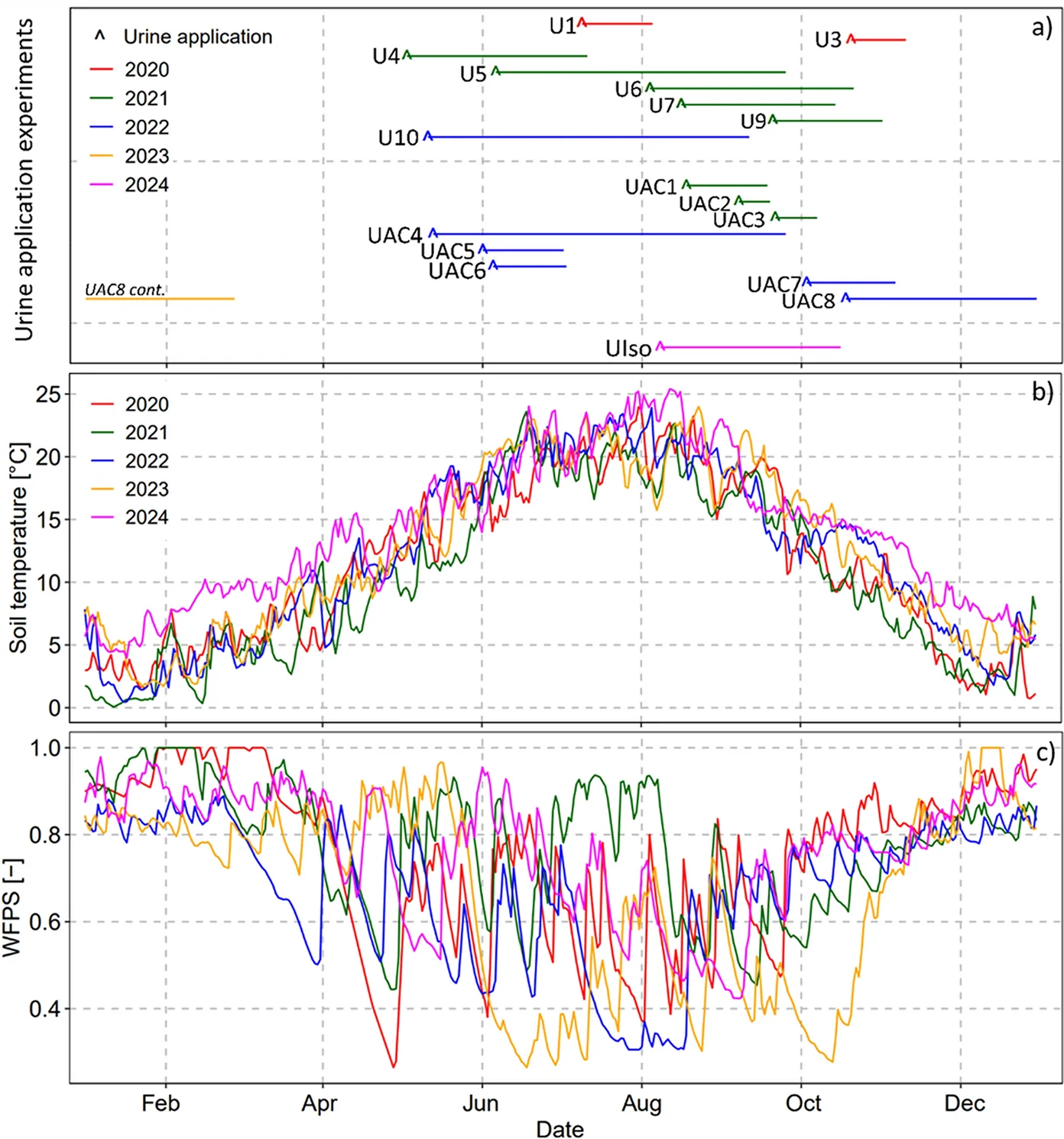
Timings of **a** urine application experiments (U1–U10 for soil mineral N sampling, UAC1–UAC8 for N_2_O flux measurements with 4-hourly temporal resolution by automatic chambers, UIso for N_2_O flux and isotopic composition measurements with automated time-integrating chambers). **b** Soil temperature at 50 mm depth and c) soil water-filled pore space WFPS at 50 mm depth over time

The length of automatic chamber experiments varied between 12 and 135 days, with five out of eight experiments lasting over 30 days (Fig. 1; Table 1). Each experiment experienced precipitation events. The soil WFPS showed large variations during the grazing season but was generally more stable in the winter (Fig. 1). Soil temperatures at 50 mm depth were lowest in UAC8 (average 6 °C), and highest in UAC4–UAC6 (average 20 °C).

**Table 1:** List of conducted automatic chamber experiments measuring N_2_O emission from urine patches F(N_2_O)_urine_ continuously by using automatic chambers including mean cumulative emissions ∑F(N_2_O)_urine_ (with standard error SE), emission factor values (EF_urine_) were only calculated for experiments longer than 30 days, the maximum flux observed and the timing of maximum flux (time since urine application t_appl_)

Experiment	Application date	Measure- ment length	∑F(N_2_O)_urine_ [mg N_2_O-N patch^−1^]		EF_urine_ [%]	Maximum flux [µg N_2_O-N patch^−1^ h^−1^]	t_appl_ at max. flux
		[days]	Mean	SE			[days]
UAC1	19.08.2021	32	113.8	14.5	0.81	759	3
UAC2	08.09.2021	12	10.3	1.2	–	167	7
UAC3	22.09.2021	16	8.2	3.1	–	135	0
UAC4	14.05.2022	135	21.0	13.0	0.28	114	0
UAC5	02.06.2022	31	22.0	0.9	0.27	358	6
UAC6	06.06.2022	28	20.0	14.0	–	374	7
UAC7	04.10.2022	34	28.7	7.9	0.27	175	9
UAC8	19.10.2022	131	46.1	10.2	0.45	351	2

**Fig. 2:**
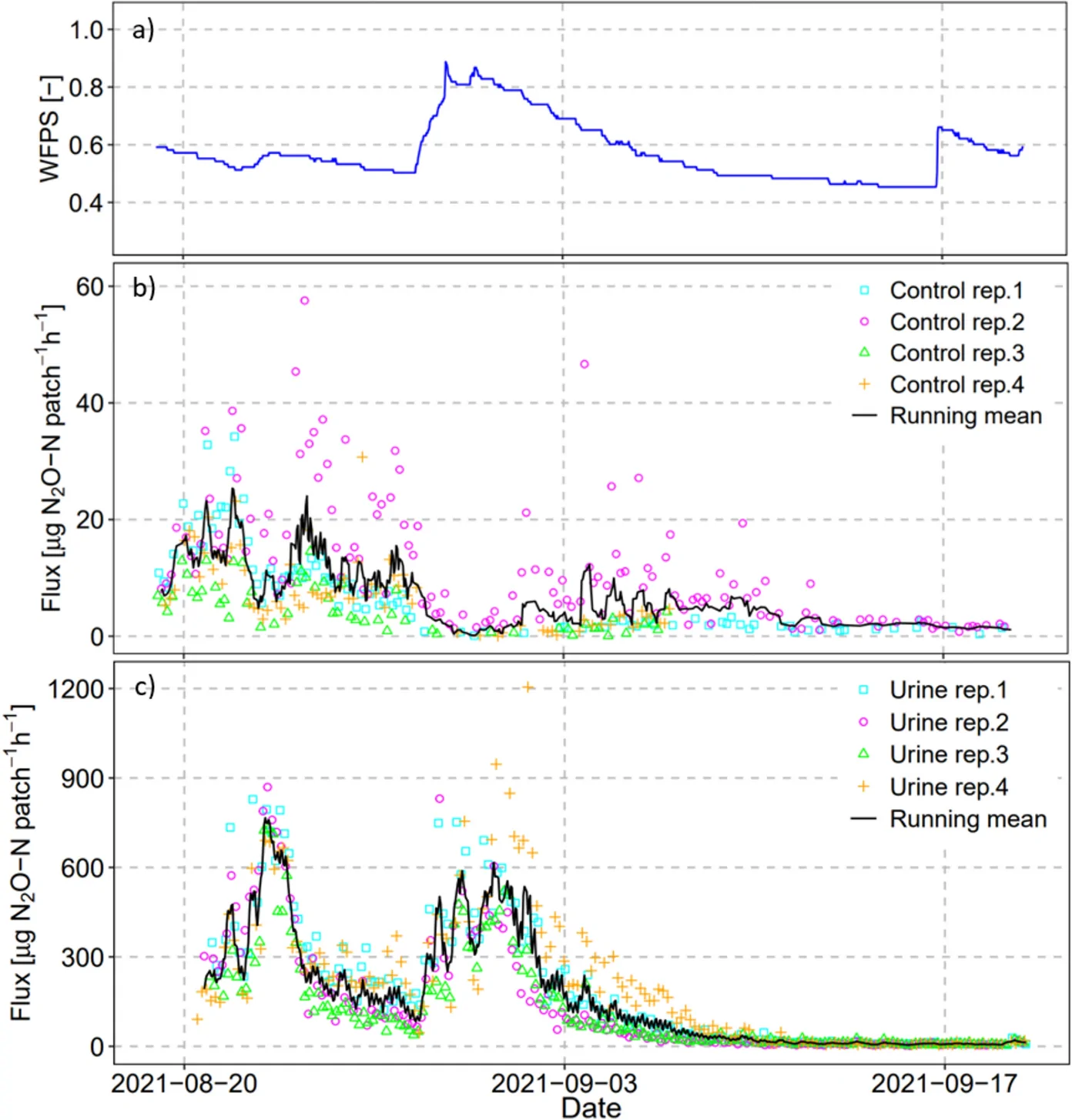
Temporal evolution and running mean of measured N_2_O fluxes from individual replicate chambers (rep.1–rep.4) of **b** the untreated control (F(N_2_O)_control_) and **c** the urine treatment (F(N_2_O)_treatment_) of experiment UAC1. Urine application was applied 20th August 2021. Note the different y-axis scales. **a** Soil water-filled pore space WFPS measured in parallel to UAC1 at 50 mm depth

Figure 2 exemplarily demonstrates the temporal evolution of measured N_2_O fluxes of the individual repetitions and the corresponding mean of the urine patch treatment F(N_2_O)_treatment_ and the untreated control F(N_2_O)_control_ in experiment UAC1. Over all experiments, F(N_2_O)_control_ ranged from 0 to 58 µg N_2_O-N patch^−1^ h^−1^, considerably lower and less dynamic than F(N_2_O)_urine_. Within the individual experiments, F(N_2_O)_urine_ were dynamically peaking up e.g. after increase of WFPS. Maximum F(N_2_O)_urine_ were observed between 0–9 days after urine application. F(N_2_O)_urine_ were highest in UAC1 reaching a maximal flux of 1200 µg N_2_O-N patch^−1^ h^−1^. In the other experiments, maximal F(N_2_O)_urine_ were between 100–718 µg N_2_O-N patch^−1^ h^−1^ (Table 1).

**Fig. 3:**
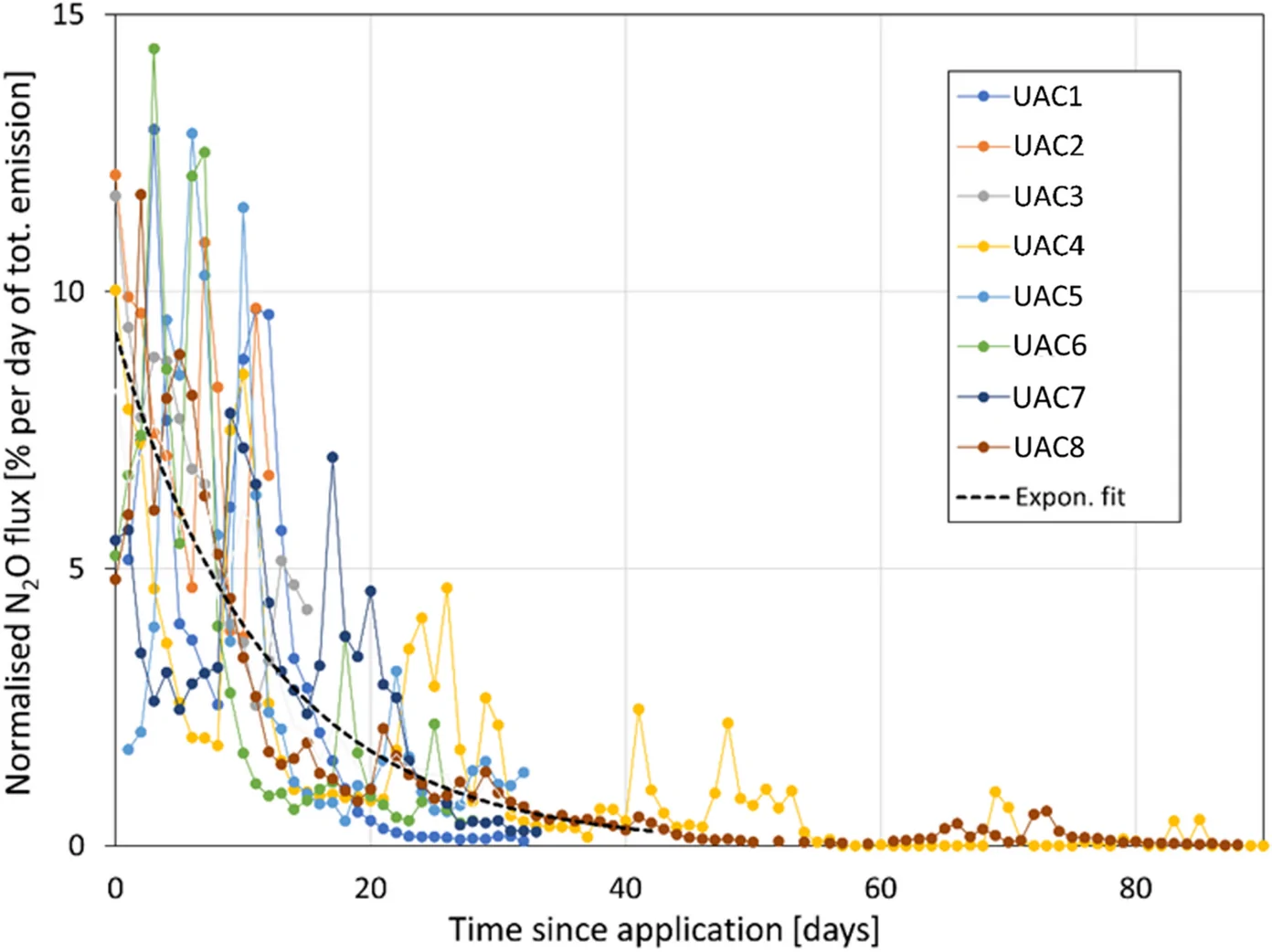
Normalised N_2_O flux as fraction [% per day] of total cumulative emissions ∑F(N_2_O)_urine_ for the different automatic chamber experiments (UAC1–UAC8) vs. the time since urine application (t_appl_) and the average exponential fit curve across all the experiments

In order to present a general overview of the temporal dynamics of all experiments, the N_2_O emissions from the urine treated plots were normalised with the total cumulative emission of the respective plot (Fig. 3) that ranged from 8.2–113.8 mg N_2_O-N patch^−1^ (Table 1). Apart from short-term variations (1–10 days) the experiments show a general exponential decay of N_2_O emission with time. The main fraction (c. 90%) of cumulative emissions were emitted within the first 30 days after urine application.

### Performance of random forest model and driver analysis

**Fig. 4:**
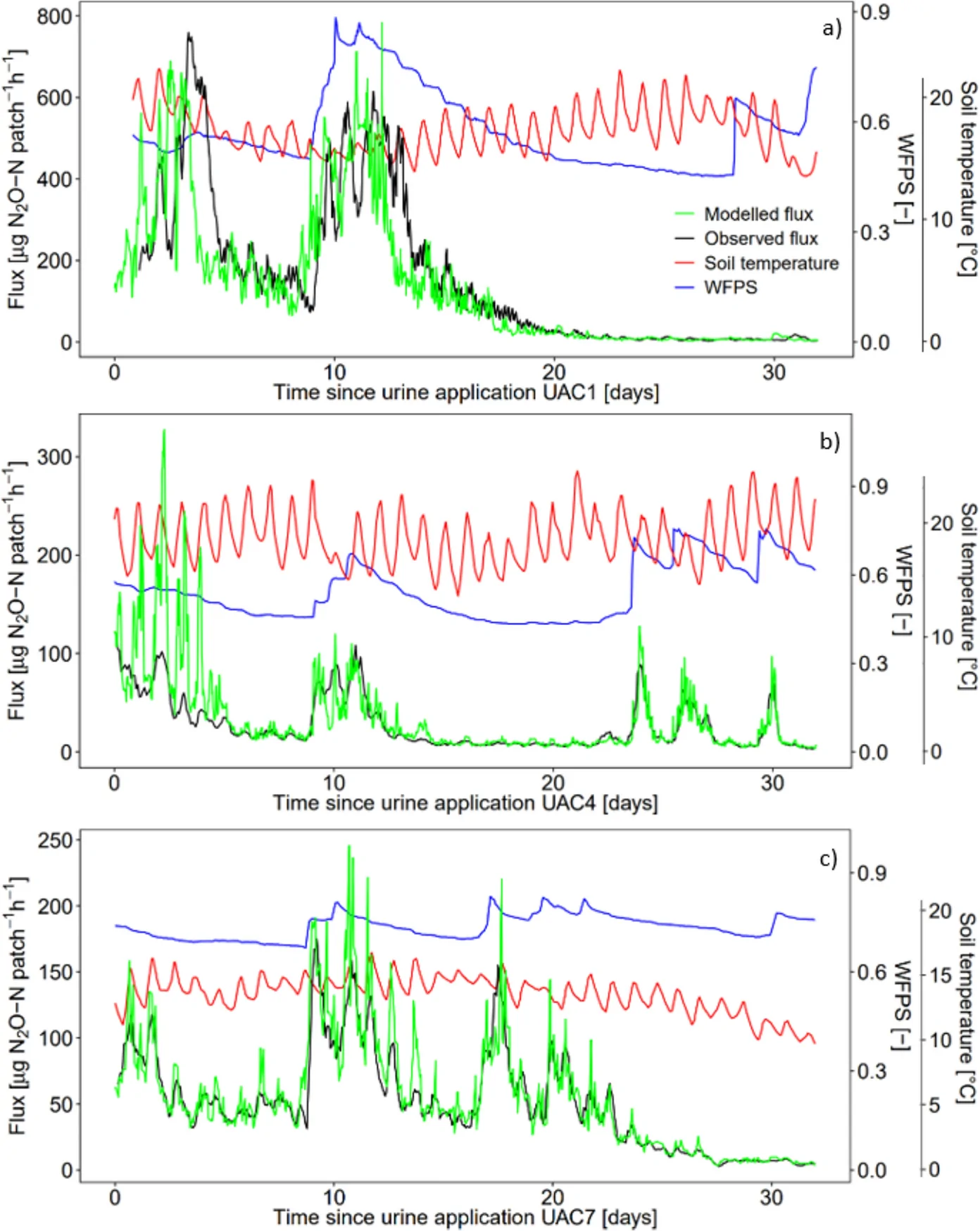
Development of observed urine N_2_O fluxes (running mean) and predictions by the random forest (RF) model within 30 days after application for experiments a) UAC1, b) UAC4 and c) UAC7, plotted together with soil water-filled pore space (WFPS) and soil temperature at 50 mm soil depth

A random Forest (RF) model was used to identify the importance of drivers on F(N_2_O)_urine_. The best agreement between observed fluxes against predictions was found for the predictors WFPS, T_s_ (soil temperature) and t_appl_ (time since urine application). Cumulative precipitation did not increase the predictive ability of the RF model. The R^2^, RMSE and MAE were 0.90, 32 µg N_2_O-N patch^−1^ h^−1^, 13 µg N_2_O-N patch^−1^ h^−1^ for the whole dataset, and 0.94, 24 µg N_2_O-N patch^−1^ h^−1^, 10 µg N_2_O-N patch^−1^ h^−1^ for the 25% validation dataset, respectively. Accordingly, the temporal evolution of F(N_2_O)_urine_ including peaks were captured generally well by the random forest model, although the diurnal variation was sometimes overestimated (Fig. 4). The examples in Fig. 4 also show that the main variations in the emission time series are related to changes in WFPS, while T_s_ explained the usually minor diurnal variations.

**Fig. 5:**
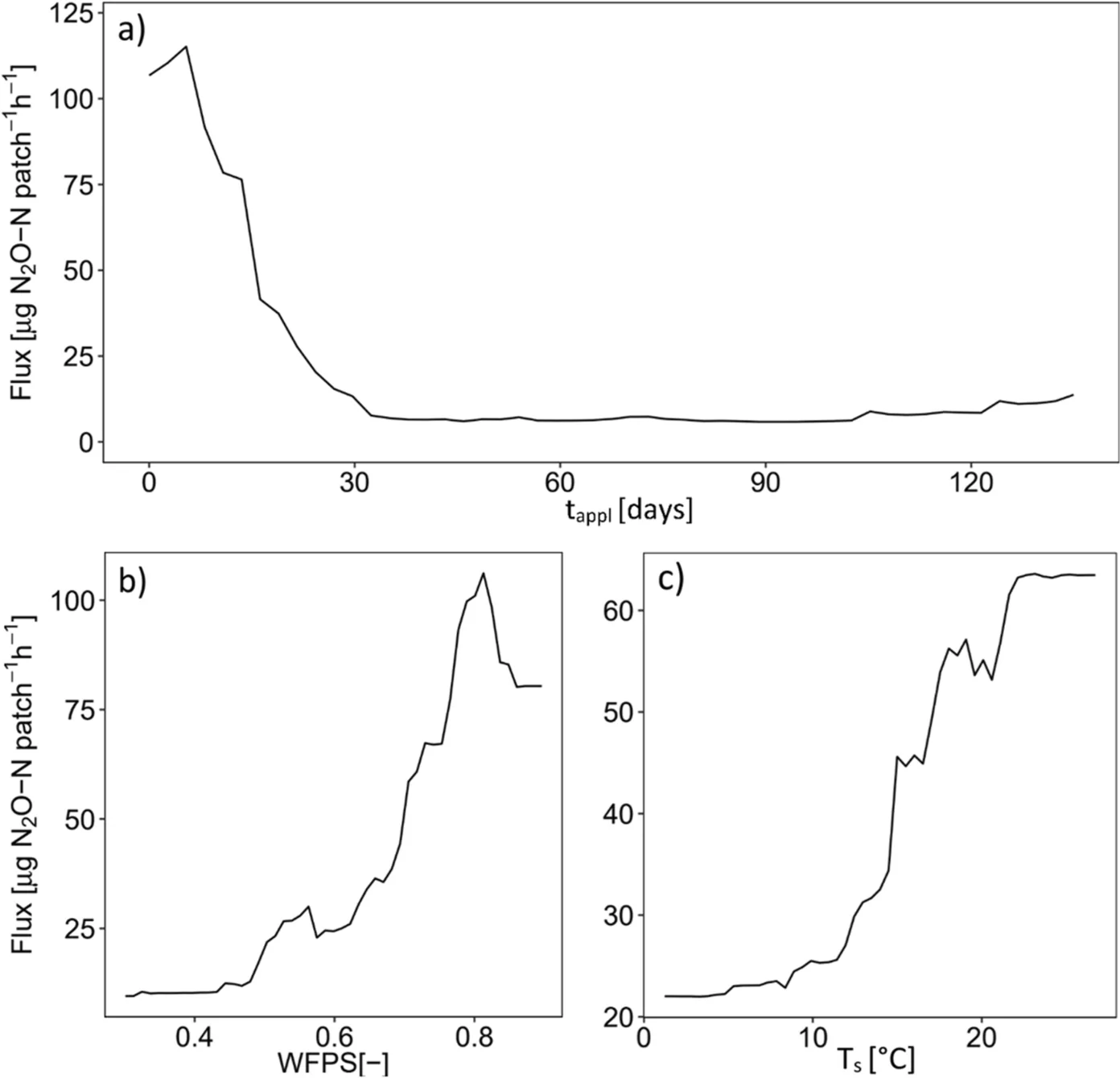
Partial dependence plots of random forest model for the N_2_O flux on the three main predictors: time since application (t_appl_), soil water-filled pore space (WFPS) and soil temperature (T_s_)

Based on the mean decrease in accuracy when randomly permuting a variable (%IncMSE), the time since urine application (t_appl_) had the greatest influence (110%). The partial dependence plot (PDP) shows maximum N_2_O emissions in the first 10 days after urine application decreasing almost linearly until reaching a constant baseline at t_appl_ = 35 d (Fig. 5a). WFPS was the second most important predictor variable (%IncMSE = 75%). In the PDP, the emissions increase almost continuously with WFPS up to 0.80 WFPS. The third predictor T_s_ had a lower impact (%IncMSE = 35%). N_2_O emissions show a steep increase around T_s_ = 15 °C until reaching a plateau at 25 °C.

### Soil mineral nitrogen

**Fig. 6:**
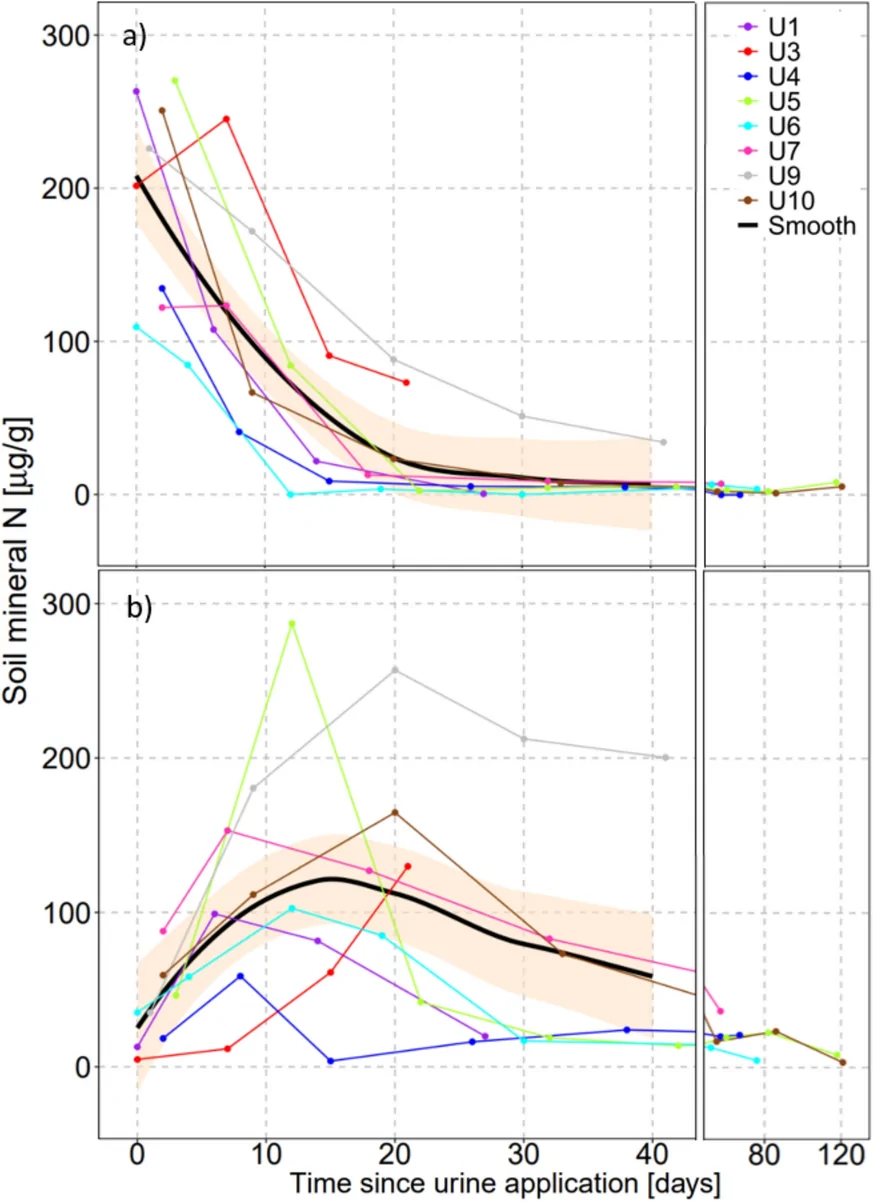
Soil mineral nitrogen (N) found as soil ammonium NH_4_^+^-N in **a** and soil nitrate NO_3_^−^-N in **b** in urine patches in 0–50 mm depth over time since urine application (t_appl_), together with fitted smooth line and 95% confidence interval. Soil mineral contents in untreated control areas were subtracted from respective urine treatments

Soil ammonium (NH_4_^+^) and nitrate (NO_3_^−^) contents of urine treatments were considerably higher than for the untreated controls (i.e. averaged 4 [µg g^−1^] NH_4_^+^-N and 10 [µg g^−1^] NO_3_^−^-N in 0–50 mm depth). For the urine treatment, the maximum soil NH_4_^+^-N contents for most experiments were observed on the first sampling day after application (t_appl_ = 1–4 d), with 109–270 [µg g^−1^]. For experiment U3 and U7 NH_4_^+^-N concentrations peaked at the second sampling day (Fig. 6; Table 2). Soil NH_4_^+^-N content levels declined with increasing t_appl_. On average, the enhanced NH_4_^+^-N pool was depleted after 30—40 d since urine application; however earlier for U6 at t_appl_ = 13 d and later (outside the sample interval) for U9. Soil NO_3_^−^-N contents in urine patches were 1-27 [µg g^−1^] on the first sampling day after urine application (t_appl_ = 1-4 d) increasing steadily up to 59-270 [µg g^−1^] NO_3_^−^-N. The maximum soil NO_3_^−^-N contents were found in a period from t_appl_ = 7 to 21 d, followed by decreasing soil NO_3_^−^-N contents, which approached zero at t_appl_ = 43-122 d. In U1, U3, U4, U7 and U9, however, NO_3_^−^-N analysis in standard urine patches was discontinued before nitrate was metabolized.

**Table 2:** List of experiments with soil mineral nitrogen (ammonium NH_4_^+^ and nitrate NO_3_^−^) sampling in urine patches after standard synthetic urine application (167 g N m^−2^). A detailed description of the experiments (U1–U10) can be found in Barczyk et al. (2023). Soil samples were taken for 0–50 mm soil depth at regular time intervals after urine application. For every experiment, the day of maximum observed NH_4_^+^ and NO_3_^−^ concentrations (with concentration values) and the day of 95% decay are listed. The mineral N contents in respective control areas were subtracted. All timing values are given as time since application t_appl_ [d]

Exp	Sampling days [d]	Concentration Maximum				95% decay time	
		NH_4_^+^-N		NO_3_^−^-N		NH_4_^+^-N	NO_3_^−^-N
		[µg g^−1^]	[d]	µg g^−1^	[d]	[d]	[d]
U1	{1, 7, 15, 28}	263 ± 26	1	99 ± 14	7	28	–
U3	{1, 8, 16, 22}	202 ± 25	8	130 ± 20	22	-	–
U4	{3, 9, 16, 27, 39, 58, 68}	135 ± 62	3	59 ± 14	9	27	–
U5	{4, 13, 23, 33, 43, 61, 83, 119}	270 ± 76	4	287 ± 18	13	23	43
U6	{1, 5, 13, 20, 31, 53, 77}	109 ± 42	1	103 ± 32	13	13	77
U7	{3, 8, 19, 33, 58}	123 ± 18	8	153 ± 24	8	19	–
U9	{2, 10, 21, 31, 42}	226 ± 51	2	257 ± 22	21	-	–
U10	{3, 10, 21, 34, 56, 87, 122}	251 ± 19	3	164 ± 10	21	34	122

### N_2_O production pathways

In one urine application experiment (UIso), starting 8 August 2024, the isotopic composition of weekly average N_2_O fluxes was analyzed. To estimate the contribution of the main N_2_O production pathways the stable isotopic composition of the emitted N_2_O was interpreted using a Monte Carlo simulation approach implemented in the FRAME model (Lewicki et al. 2022). The model accounts for differences in isotopic composition of the considered production pathways and isotopic fractionation during partial N₂O reduction (Fig. 9). The modelled results (Table 3) reveal a temporal pattern in which the relative contributions of nitrification were highest after urine application and then decreased as bacterial denitrification, and nitrifier denitrification contributed a larger share of N_2_O emissions. During the initial high-flux period (08–12 August), nitrification accounted for ~ 63% of the total N_2_O emissions, whereas bacterial denitrification and nitrifier denitrification contributed only ~ 8% and ~ 27%, respectively. Between the 12th and 27th of August, the contribution of nitrification declined sharply to 12–27%, while bacterial denitrification and nitrifier denitrification increased their relative proportions to ~ 41% and 47%, respectively. In the later season (mid-September to mid-October), N_2_O fluxes were low, but the relative contributions of the various production pathways fluctuated with nitrifier denitrification contributing ~ 31–58%, bacterial denitrification ~ 29–38%, and nitrification ~ 4–37%. Such oscillations in the relative contributions of nitrifier denitrification, bacterial denitrification and nitrification indicate that the balance between the various main N_2_O production pathways is highly sensitive to temporal changes in soil physicochemical conditions, such as substrate availability, soil moisture, and aeration.

**Table 3:** Relative contributions of nitrifier denitrification (nD), bacterial denitrification (bD) and nitrification (Ni) to N_2_O emissions after artificial urine application (mean and 95% CI). Results were estimated using the FRAME model and include a correction for isotopic enrichment due to microbial N_2_O reduction

Time period	N_2_O emissions	Relative contributions		
	[mg patch^−1^ d^−1^]	nD	bD	Ni
08.08–12.08	1.88	0.27 [0.02–0.67]	0.08 [0.00–0.31]	0.63 [0.28–0.91]
12.08–19.08	1.34	0.40 [0.03–0.63]	0.34 [0.02–0.78]	0.27 [0.04–0.49]
19.08–27.08	0.41	0.47 [0.03–0.70]	0.41 [0.02–0.87]	0.12 [0.01–0.26]
27.08–03.09	0.22	na^a^	na^a^	na^a^
03.09–12.09	0.17	na^a^	na^a^	na^a^
12.09–18.09	0.27	na^a^	na^a^	na^a^
18.09–25.09	0.19	0.43 [0.04–0.66]	0.30 [0.01–0.73]	0.27 [0.07–0.49]
25.09–02.10	0.15	0.31 [0.02–0.57]	0.33 [0.00–0.84]	0.37 [0.01–0.92]
02.10–09.10	0.15	na^a^	na^a^	na^a^
09.10–16.10	0.14	na^a^	na^a^	na^a^
16.10–24.10	0.10	na^a^	na^a^	na^a^
24.10–31.10	0.08	na^a^	na^a^	na^a^
31.10–07.11	0.29	na^a^	na^a^	na^a^
07.11–16.11	1.65	0.58 [0.11–0.80]	0.38 [0.01–0.85]	0.04 [0.00–0.11]

^a^no N_2_O isotopic source signature was determined for this data as low flux conditions did not pass the set quality criteria

### Effect of manual chamber sampling regimes on observed emissions

**Fig. 7:**
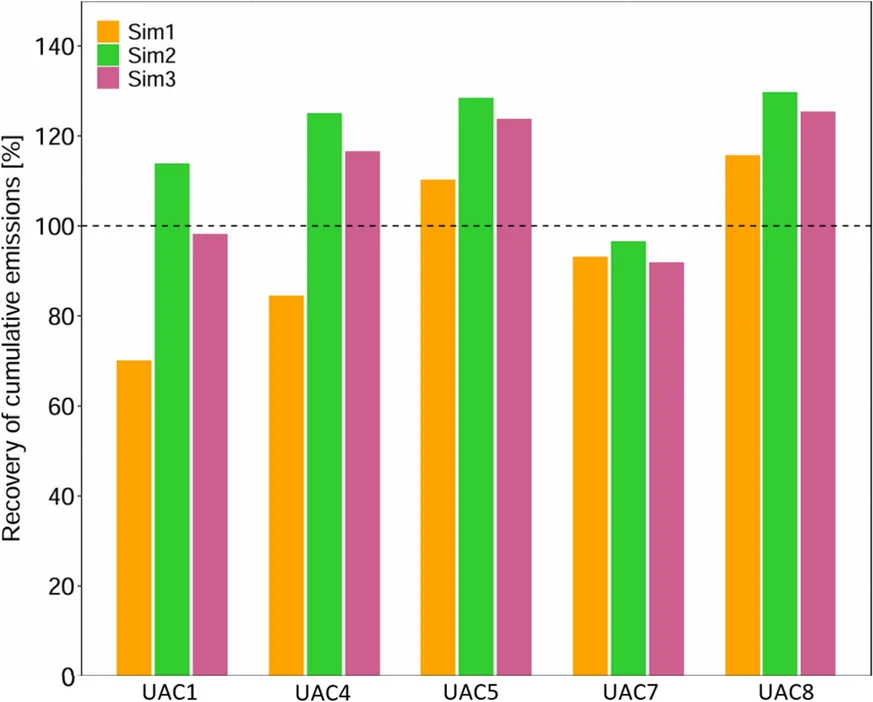
Recovery of cumulative fluxes ∑F(N_2_O)_urine_ derived from automated chamber measurements when simulating three manual chamber flux sampling regimes (Sim1, Sim2 Sim3). This comparison is performed for automated chamber experiments (UAC) with experimental lengths > 30 days

**Fig. 8:**
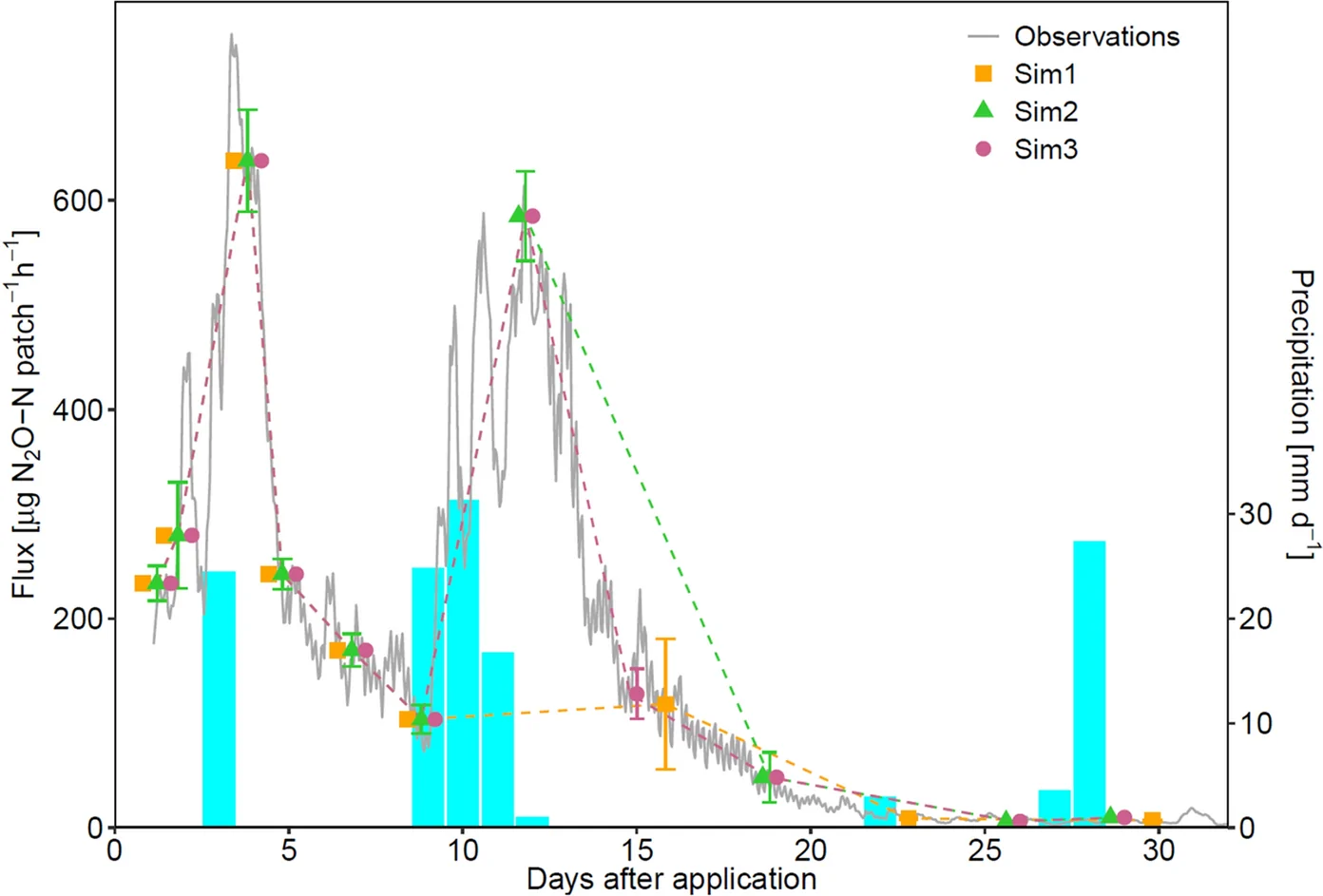
Development of observed high-resolution N_2_O fluxes (mean of 3 replicate automated chambers) in experiment UAC1 in comparison to simulated manual chamber sampling regimes (Sim1, Sim2 Sim3). Simulated manual chamber measurements are linearly interpolated (dashed lines). Precipitation per day (cyan bars) relates to the second y-axis

**Fig. 9:**
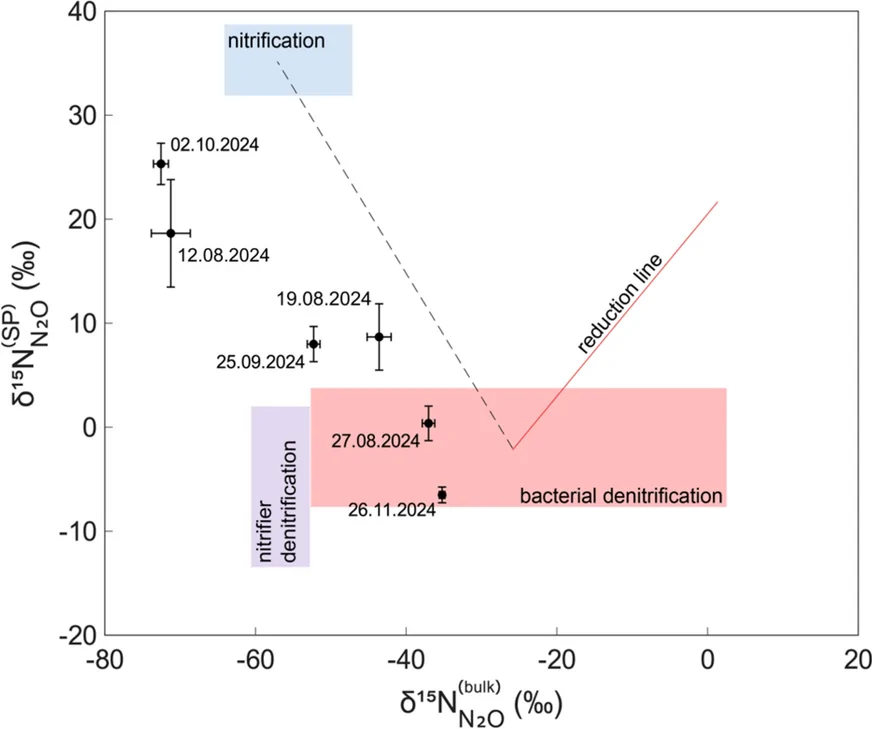
Microbial isotopic source signature of the N_2_O fluxes in the UIso experiment displayed as plot of δ^15^N^SP^ against δ^15^N.^bulk^ (black dots with 95%-CI, labeled with the end date of the sampling time period) to interpret the relative contribution of nitrification, nitrifier denitrification and bacterial denitrification (see Table 3). The colored areas represent ranges of isotopic values reported in the literature for the respective processes (Yu et al. 2020)

The recovery of simulated compared to observed (“true”) cumulative emissions was 70% to 116% for Sim1, 97% to 130% for Sim2, and 92% to 125% for Sim3 (Fig. 7). For Sim1, the bias was highest in UAC1 (-30%). Figure 8 exemplary shows the evolution of F(N_2_O)_urine_ in UAC1, demonstrating that the manual sampling in Sim1 could not capture a significant emission peak occurring at t_appl_ = 9–13 d in contrast to Sim2 and Sim3. Furthermore, in UAC4, a significant emission peak at t_appl_ = 9–12 d was not captured by Sim1, but it was by Sim2 and Sim3. In UAC5 and UAC8, ∑F(N_2_O)_urine_sim_ were higher than ∑F(N_2_O)_urine_ for all of three simulations. On average, the bias for Sim1 was − 5% while for Sim2 it was + 19%, and for Sim3 it was + 11%.

## Discussion

### Drivers of N_2_O emission dynamics

Automatic flux chamber measurements showed highly variable F(N_2_O)_urine_ that are in the range of reported flux values (e.g. Krol et al. 2016; Chadwick et al. 2018). The RF algorithm found that the predictor t_appl_ had the largest impact on F(N_2_O)_urine_. The PDP for predictor t_appl_ shows highest F(N_2_O)_urine_ values shortly after urine application decreasing continuously until t_appl_ = 35 d (Fig. 5a). This is consistent with the observation that 90% of total N_2_O emissions were, on average, emitted within 30 days after urine application (Fig. 3). Also other studies (e.g. van Groeningen et al. 2005; Bell et al. 2015; Cardenas et al. 2016; Barczyk et al. 2023) confirm that the major part of N_2_O emissions occur within 30 days after urine application.

In our study, the application of urine caused an increased supply of mineral N in the soil of up to 270 µg g^−1^ NH_4_^+^-N (Fig. 6). Maximum amounts of NH_4_^+^-N occur shortly after urine application and the temporal trend of NH_4_^+^-N shows an exponential decrease comparable to the PDP of the predictor t_appl_ (Fig. 5a). This suggests that microbial processes using NH_4_^+^-N as feed substrate, like nitrification or nitrifier denitrification could represent a major contribution to N_2_O production. Soil NO_3_^−^-N contents increased in parallel to decreasing soil NH_4_^+^-N contents, thus bacterial denitrification from urine N could already occur at t_appl_ = 1 d (see Fig. 6b). This transition from nitrification to denitrification dominated N_2_O emissions was confirmed by stable isotope analysis. In fact, nitrification contributed a major share of N_2_O emissions in the first 4-day period after urine application, while denitrification dominated afterwards at reduced emission fluxes (see Table 3). Carter (2007) found an equal contribution of nitrification and bacterial denitrification to N_2_O emission from urine patches at an intermediate soil WFPS of 0.45 during 24 h past urine application. In Ambus et al. (2007), bacterial denitrification was the sole source for F(N_2_O)_urine_ after 12 days of incubation. Koops et al. (1997), however, concluded that nitrification was the main N_2_O producing process in a dry grassland peat soil after urine application because N_2_O production through bacterial denitrification was significant only after a rainfall event leading to a high WFPS. The initial dominance of nitrification (see Table 3) observed in our study was possibly stimulated by the high soil NH_4_^+^-N pool and by increased soil pH after urea hydrolysis (Dancer et al. 1973; Somda et al. 1997), while initial denitrification was possibly affected by wetting the soil through urine application (Koops et al. 1997). Anaerobic conditions, an elevated soil pH and/or a high soil NH_4_^+^ pool due to urine addition also favor N_2_O production through nitrifier denitrification (Wrage-Mönnig et al. 2018; Clough et al. 2020). In our study, the temporal development of N_2_O fluxes (Fig. 3) was not positively correlated to the soil NO_3_^−^-N content, but to soil NH_4_^+^-N content (Fig. 6a), therefore nitrifier denitrification may be more predominant than bacterial denitrification. This is in agreement with the isotopic results in Table 3, which show that the estimated contribution of nitrifier denitrification to N_2_O emissions often exceeded bacterial denitrification. However, a clear distinction between nitrifier denitrification and bacterial denitrification is difficult based on the available isotopic composition data (see CI in Table 3).

Microbial N_2_O forming pathways are often related to the soil aeration status, assuming that nitrification rather occurs under aerobic conditions and denitrification under anaerobic conditions (Butterbach-Bahl 2013). In our isotopic experiment, the contribution of nitrification and denitrification to measured N_2_O emissions could not be linked to the soil WFPS (not shown here), probably because it was a cumulative N_2_O measurement over one week with variable WFPS during the week, or the co-occurrence of N_2_O transformation pathways. In the high-frequency automatic chamber experiments, however, soil WFPS showed large variations across seasons (e.g. ranging from 0.30 to 0.81 during UAC4) and F(N_2_O)_urine_ increased with increasing WFPS (see examples in Fig. 4). Only experiment UAC8 lasting over winter, gave low F(N_2_O)_urine_ at constantly high WFPS (average of 0.8) possibly explained by low microbial activities at soil temperatures < 5 °C.

The timespan of increased soil NH_4_^+^-N, and thus the source of F(N_2_O)_urine_ via nitrification and nitrifier denitrification, was depleted on average after 30–40 days. In U3, however, the decrease of soil NH_4_^+^-N and increase of NO_3_^−^-N was less pronounced in contrast to the other experiments. In this experiment, urine application was in late autumn at initial T_s_ around 10 °C and a constantly high WFPS level > 0.8. Probably both nitrification and denitrification pathways were less active at low T_s_ and high WFPS. In contrast, the initial NH_4_^+^-N pool size was lower and the decrease of NH_4_^+^-N was faster in U4 and U6 than in the other experiments. The urine was applied at a very high WFPS of 0.9. Thus, it may be that some of the urine N was leached to the subsoil during application. Furthermore, WFPS level dropped down in the next days reaching optimal conditions for nitrification.

In five out of eight experiments, we could not define the time at which the increased soil NO_3_^−^-N was depleted (Table 2) because the observation periods were too short. For experiments U5, U6 and U10, however, the high soil NO_3_^−^-N in urine patches was depleted after t_appl =_ 33–77 days, and thus also the urine-N N_2_O source. Compared to the other experiments, the NO_3_^−^-N pool was still very high at t_appl =_ 42 days in U9. A reason for this could be the absence of intensive rainfall events (see Barczyk et al. (2023)), and thus a low risk of NO_3_^−^ leaching (Steele et al 1984; Talbot et al. 2021). Furthermore, experiment U9 was conducted at the end of the grazing season with decreasing plant growth and microbial activity due to decreasing temperatures (Stres et al. 2008; Punia et al. 2020). In U5, the NO_3_^−^-N pool fell by 245 [µg g^−1^] within 11 days possibly due to NO_3_^−^ leaching after an intensive rainfall event (i.e., 20 mm rainfall in 12 h).

### Manual chamber sampling regime

Automated chamber experiments were conducted to measure F(N_2_O)_urine_ at a high temporal resolution. The main part of the emissions occurred in t_appl_ = 0–30 d. The length of this period corresponds well with measurements on F(N_2_O)_urine_ in other studies (e.g. Singh et al. 2009; Selbie et al. 2014; Sordi et al. 2014; Voglmeier et al. 2019). Also, manual chamber measurements conducted at the same experimental site showed a similar average decay of F(N_2_O)_urine_ over time (Barczyk et al. 2023).

However, our automated chamber measurements revealed highly variable F(N_2_O)_urine_ e.g. after changes in WFPS. We questioned if a manual chamber sampling regime can quantify ∑F(N_2_O)_urine_ adequately, because time gaps may be too long and emission peaks after episodic events such as rainfall may be missed. Several different sampling regimes have been used in the literature to measure N_2_O emissions from urine patches with manual chambers. The sampling regime described by Krol et al. (2016) is more or less consistent with the sampling regime Sim1 used here, with three sampling measurements in the first week after urine application, two sampling measurements in the second week, followed by once per week, once every two weeks until day 24, and once a month until the end of the experiment. In Chadwick et al. (2018) the sampling frequency was higher, with 4–5 times in the first week, 4–5 times in the second week, 2 times per week for the next two weeks, and then once per week for 1 month. Singh et al. (2009) measured daily for the first week, then every second day for two weeks, twice per week for one week, and once per week for the remaining period until F(N_2_O)_urine_ approached background levels.

In our study, simultaneous manual and automatic chamber measurements in UAC1 demonstrated a reasonable agreement between the two chamber methods. Furthermore, the temporal dynamic of F(N_2_O)_urine_ could be captured by manual chamber measurements (Fig. 8) resulting in similar ∑F(N_2_O)_urine_ and EF_urine_. In other situations, however, this might not be the case. We simulated three schedules of manual chamber measurements. The Sim1 regime resulted in an average underestimation of cumulated emissions of only 5%, while Sim2 and Sim3 resulted in an overestimation of + 19% and + 11%. In most cases, Sim1 was not able to capture enhanced F(N_2_O)_urine_ after rainfall at t_appl_ > 8 d. Sim2 and Sim3, on the other hand, usually captured enhanced F(N_2_O)_urine_ after rainfall, which caused an overestimation of interpolated F(N_2_O)_urine_ if the captured emission peak was high and of short duration (e.g. 1 day). Additional sampling three days after intensive rainfall in Sim3 slightly diminished the overestimation of Sim2, as Sim3 better grasped the decline of the N_2_O peak. Accordingly, van der Weerden et al. (2013) found similar biases of -3% to + 21% for 3-day manual chamber sampling intervals on urine patches, which were reduced slightly to between -3% and + 18% when measuring specifically after intensive rainfall events > 10 mm d^−1^.

## Conclusions

Our study aimed at understanding the temporal dynamics of N_2_O emissions from urine patches and to identify drivers and major production pathways. In contrast to manual chamber measurements, automatic chambers offered more frequent flux measurements in a cycle of 4 h. The spatial application, however, was more limited. According to the applied random forest algorithm, the time since urine application (t_appl_) was the strongest driver for F(N_2_O)_urine_. Its influence showed a decreasing course until approximately t_appl_ = 35 d, similar to the course of the soil NH_4_^+^ content. This suggests that nitrification and / or nitrifier denitrification might be dominant contributors to ∑F(N_2_O)_urine_. Stable isotopic composition of the emitted N_2_O indicates a major contribution of nitrification during the initial high N_2_O flux period and dominance of denitrifying pathways in subsequent periods. A further distinction between nitrifier denitrification and bacterial denitrification could be addressed using dedicated isotope labelling experiments but was beyond the scope of this study. Further studies examining the contribution of individual microbial pathways to F(N_2_O)_urine_ under varying environmental conditions would help to better understand the dynamic of F(N_2_O)_urine_.

In addition, the observed 4-h flux time series were used for the simulation of a manual chamber sampling strategies as used in Barczyk et al. (2023) at the same site. The results show that, on average over multiple experiments, the cumulative N_2_O emission can be reproduced to a reasonable degree by manual chamber measurements (bias of − 5% in Sim1) with a prescribed sampling schedule independent of rainfall events.

## Data Availability

All data supporting the findings of this study are available within the paper and can be additionally provided upon request.

## Appendix

See Table

**Table 4:** Timing and duration of urine application experiments; U1–U10 for soil mineral N sampling, UAC1–UAC8 for N_2_O flux measurements with 4-hourly temporal resolution by automatic chambers, UIso for N_2_O flux and isotopic composition measurements with automated time-integrating chambers

Exp	Measurement purpose	Application date	Duration [days]
U1	Soil NH_4_^+^/NO_3_^−^	09.07.2020	29
U2	other^1^		
U3	Soil NH_4_^+^/NO_3_^−^	20.10.2020	23
U4	Soil NH_4_^+^/NO_3_^−^	06.05.2021	69
U5	Soil NH_4_^+^/NO_3_^−^	10.06.2021	120
U6	Soil NH_4_^+^/NO_3_^−^	06.08.2021	78
U7	Soil NH_4_^+^/NO_3_^−^	19.08.2021	59
U8	other^1^		
U9	Soil NH_4_^+^/NO_3_^−^	22.09.2021	43
U10	Soil NH_4_^+^/NO_3_^−^	14.05.2022	123
UAC1	N_2_O fluxes	19.08.2021	32
UAC2	N_2_O fluxes	08.09.2021	12
UAC3	N_2_O fluxes	22.09.2021	16
UAC4	N_2_O fluxes	14.05.2022	135
UAC5	N_2_O fluxes	02.06.2022	31
UAC6	N_2_O fluxes	06.06.2022	28
UAC7	N_2_O fluxes	04.10.2022	34
UAC8	N_2_O fluxes	19.10.2022	131
UIso	N_2_O fluxes + Isotopic signature	08.08.2024	100

^1^The naming of experiments U1-U10 was used consistently with Barczyk et al. (2023). The experiments U2 and U8 did not include soil NH4 + /NO3^−^ analysis and were therefore not used in the present study

4 and Fig. 9.

## References

[CR1] Ambus P, Petersen SO, Soussana J-F (2007) Short-term carbon and nitrogen cycling in urine patches assessed by combined carbon-13 and nitrogen-15 labelling. Agric Ecosyst Environ 121:84–92

[CR2] Ammann C, Neftel A, Jocher M, Fuhrer J, Leifeld J (2020) Effect of management and weather variations on the greenhouse gas budget of two grasslands during a 10-year experiment. Agric Ecosyst Environ 292:106814. 10.1016/j.agee.2019.106814

[CR3] Barczyk L, Kuntu-Blankson K, Calanca P, Six J, Ammann C (2023) N_2_O emission factors for cattle urine: efect of patch characteristics and environmental drivers. Nutr Cycl Agroecosyst 127:173–189. 10.1007/s10705-023-10290-0 PMC10576711 37846228

[CR101] Barczyk L, Six J, Ammann C (2024) Partitioning and driver analysis of eddy covariance derived N2O emissions from a grazed and fertilized pasture. Agri Forest Meteorol 359:110278. 10.1016/j.agrformet.2024.110278

[CR4] Bell MJ, Rees RM, Cloy JM, Topp CFE, Bagnall A, Chadwick DR (2015) Nitrous oxide emissions from cattle excreta applied to a Scottish grassland: effects of soil and climatic conditions and a nitrification inhibitor. Sci Total Environ 508:343–353 10.1016/j.scitotenv.2014.12.008 25497356

[CR5] Braker G, Conrad R (2011) Diversity, structure, and size of N_2_O-producing microbial communities in soils - what matters for their functioning? Adv Appl Microbiol 75:33–70. 10.1016/B978-0-12-387046-9.00002-5 21807245

[CR6] Bussink DW, Oenema O (1998) Ammonia volatilization from dairy farming systems in temperate areas: a review. Nutr Cycl Agroecosyst 51:19–33

[CR7] Butterbach-Bahl K, Baggs EM, Dannenmann M, Kiese R, Zechmeister-Boltenstern S (2013) Nitrous oxide emissions from soils: how well do we understand the processes and their controls? Philos Trans R Soc Lond B Biol Sci 368:20130122. 10.1098/rstb.2013.0122 PMC3682742 23713120

[CR8] Cabrera ML, Kissel DE, Bock BR (1991) Urea hydrolysis in soil: effects of urea concentration and soil pH. Soil Biol Biochem 23:1121–1124

[CR9] Cai Y, Akiyama H (2016) Nitrogen loss factors of nitrogen trace gas emissions and leaching from excreta patches in grassland ecosystems: a summary of available data. Sci Total Environ 572:185–195. 10.1016/j.scitotenv.2016.07.222 27498380

[CR10] Cardenas LM, Misselbrook TM, Hodgson C, Donovan N, Gilhespy S, Smith KA, Dhanoa MS, Chadwick D (2016) Effect of the application of cattle urine with or without the nitrification inhibitor DCD, and dung on greenhouse gas emissions from a UK grassland soil. Agric Ecosyst Environ 235:229–241. 10.1016/j.agee.2016.10.025 PMC5142719 27974862

[CR11] Carter MS (2007) Contribution of nitrification and denitrification to N_2_O emissions from urine patches. Soil Biol Biochem 39:2091–2102

[CR12] Chadwick DR, Cardenas L, Misselbrook TH, Smith KA, Rees RM, Watson CJ, McGeough KL, Williams JR, Cloy JM, Thorman RE, Dhanoa MS (2014) Optimizing chamber methods for measuring nitrous oxide emissions from plot-based agricultural experiments. Eur J Soil Sci 65:295–307

[CR13] Chadwick DR, Cardenas LM, Dhanoa MS, Donovan N, Misselbrook T, Williams JR, Thorman RE, McGeough KL, Watson CJ, Bell M, Anthony SG, Rees RM (2018) The contribution of cattle urine and dung to nitrous oxide emissions: quantification of country specific emission factors and implications for national inventories. Sci Total Environ 635:607–617. 10.1016/j.scitotenv.2018.04.152 PMC6024564 29679833

[CR14] Charteris AF, Chadwick DR, Thorman RE, Vallejo A, de Klein CAM, Rochette P, Cardenas LM (2020) Global research alliance N_2_O chamber methodology guidelines: recommendations for deployment and accounting for sources of variability. J Environ Qual 49:1092–1109 10.1002/jeq2.20126 33016455

[CR15] Clough TJ, Cardenas LM, Friedl J, Wolf B (2020) Nitrous oxide emissions from ruminant urine: science and mitigation for intensively managed perennial pastures. Curr Opin Env Sust 45:21–27. 10.1016/j.cosust.2020.07.001

[CR16] Dancer WS, Peterson LA, Chesters G (1973) Ammonification and nitrification of N as influenced by soil pH and previous N treatments. Soil Sci Soc Am J 37:67–69

[CR17] Dangal SR, Tian H, Xu R, Chang J, Canadell JG, Ciais P, Pan S, Yang J, Zhang B (2019) Global nitrous oxide emissions from pasturelands and rangelands: magnitude, spatiotemporal patterns, and attribution. Global Biogeochem Cycles 33:200–222

[CR18] de Klein CAM, Harvey MJ, Clough TJ, Petersen SO, Chadwick DR, Venterea RT (2020) Global research alliance N_2_O chamber methodology guidelines: introduction, with health and safety considerations. J Environ Qual 49:1073–1080. 10.1002/jeq2.20131 33016437

[CR19] Dijkstra J, Oenema O, Van Groenigen JW, Spek JW, Van Vuuren AM, Bannink A (2013) Diet effects on urine composition of cattle and N_2_O emissions. Animal 7:292–302 10.1017/S1751731113000578 23739471

[CR20] Dobbie KE, McTaggart IP, Smith K (1999) Nitrous oxide emissions from intensive agricultural systems: variations between crops and seasons, key driving variables, and mean emission factors. J Geophys Res 104:26891–26899

[CR21] Firkins JL, Reynolds C (2005) Whole-animal nitrogen balance in cattle. In: Pfeffer E, Hristov AN (eds) Nitrogen and phosphorus nutrition of cattle. CAB International, Wallingford, pp 167–186

[CR22] Flechard CR, Neftel A, Jocher M, Ammann C, Fuhrer J (2005) Bi-directional soil/atmosphere N_2_O exchange over two mown grassland systemswith contrasting management practices. Glob Chang Biol 11:2114–2127. 10.1111/j.1365-2486.2005.01056.x 34991287

[CR23] Grace PR, van der Weerden TJ, Rowlings DW, Scheer C, Brunk C, Kiese R, Butterbach-Bahl K, Rees RM, Robertson GP, Skiba UM (2020) Global research alliance N_2_O chamber methodology guidelines: considerations for automated flux measurement. J Environ Qual 49:1126–1140. 10.1002/jeq2.20124 33016438

[CR24] Harris E, Diaz-Pines E, Stoll E, Schloter M, Schulz S, Duffner C, Li K, Moore KL, Ingrisch J, Reinthaler D, Zechmeister-Boltenstern S, Glatzel S, Brüggemann N, Bhan M (2021) Denitrifying pathways dominate nitrous oxide emissions from managed grassland during drought and rewetting. Sci Adv 7:eabb7118 10.1126/sciadv.abb7118 PMC7864578 33547069

[CR25] Havsteen JC, Fatima M, Brunamonti S, Pogány A, Hausmaninger T, Wolf B, Well R, Mohn J (2025) Correction and calibration protocol for isotope data via CRDS: a study case for N2O and other isotope systems, EGUsphere, [preprint], 10.5194/egusphere-2025-4954

[CR26] Haynes RJ, Williams PH (1993) Nutrient cycling and soil fertility in the grazed pasture ecosystem. Adv Agron 46:119–199. 10.1016/S0065-2113(08)60794-4

[CR27] Hubbard RK, Newton GL, Hill GM (2004) Water quality and the grazing animal. J Anim Sci 82:E255–E263 10.2527/2004.8213_supplE255x 15471806

[CR28] Hüppi R, Felber R, Krauss M, Six J, Leifeld J, Fuss R (2018) Restricting the nonlinearity parameter in soil greenhouse gas flux calculation for more reliable flux estimates. PLoS ONE. 10.1371/journal.pone.0200876 PMC6062054 30048522

[CR29] IPCC (2006) 2006 IPCC Guidelines for National Greenhouse Gas Inventories, Prepared by the National Greenhouse Gas Inventories Programme. Eggleston HS, Buendia L, Miwa K, Ngara T., Tanabe K. (ed) Published: IGES, Japan.

[CR30] IPCC (2023) Climate Change 2023: Synthesis Report. Contribution of Working Groups I, II and III to the Sixth Assessment Report of the Intergovernmental Panel on Climate Change [Core Writing Team, H. Lee and J. Romero (eds.)]. IPCC, Geneva, Switzerland, 184 pp., 10.59327/IPCC/AR6-9789291691647

[CR31] Kool DM, Hoffland E, Hummelink EWJ, Van Groenigen JW (2006) Increased hippuric acid content of urine can reduce soil N_2_O fluxes. Soil Biol Biochem 38:1021–1027. 10.1016/j.soilbio.2005.08.017

[CR32] Koops JG, Van Beusichem ML, Oenema O (1997) Nitrous oxide production, its source and distribution in urine patches on grassland on peat soil. Plant Soil 191:57–65

[CR33] Krol DJ, Carolan R, Minet E, McGeough KL, Watson CJ, Forrestal PJ, Lanigan GJ, Richards KG (2016) Improving and disaggregating N_2_O emission factors for ruminant excreta on temperate pasture soils. Sci Total Environ 568:327–338. 10.1016/j.scitotenv.2016.06.016 27300566

[CR34] Laville P, Bosco S, Volpi I, Virgili G, Neri S, Continanza D, Bonari E (2017) Temporal integration of soil N_2_O fluxes: validation of IPNOA station automatic chamber prototype. Environ Monit Assess 189:1–21 10.1007/s10661-017-6181-2 28871518

[CR35] Lemke R, Baron V, Iwaasa A, Farrell R, Schoenau J (2012) Quantifying nitrous oxide emissions resulting from animal manure on pasture, range and paddock by grazing cattle in Canada. Final report submitted to the greenhouse gas division, environment Canada, by Agriculture and Agri-Food Canada, Saskatoon (SK).

[CR36] Lewicki MP, Lewicka-Szczebak D, Skrzypek G (2022) FRAME—Monte Carlo model for evaluation of the stable isotope mixing and fractionation. PLoS ONE 17:e0277204. 10.1371/journal.pone.0277204 PMC9704640 36441721

[CR37] Luo J, Sun XZ, Pacheco D, Ledgard SF, Lindsey SB, Hoogendoorn CJ, Wise B, Watkins NL (2015) Nitrous oxide emission factors for urine and dung from sheep fed either fresh forage rape (*Brassica napus* L.) or fresh perennial ryegrass (*Lolium perenne* L.). Animal 9:534–543 10.1017/S1751731114002742 25407839

[CR38] MeteoSwiss (2022) https://www.meteoswiss.admin.ch/climate/the-climate-of-switzerland/climate-normals/climate-diagrams-and-normals-per-station.html. Accessed 24 November 2022

[CR39] Mohn J, Biasi C, Bodé S, Boeckx P, Brewer PJ, Eggleston S, Geilmann H, Guillevic M, Kaiser J, Kantnerová K (2022) Isotopically characterised N_2_O reference materials for use as community standards. Rapid Commun Mass Spectrom 36(13):e9296 10.1002/rcm.9296 PMC9286586 35289456

[CR100] Moraes LE, Burgos SA, DePeters EJ, Zhang R, Fadel JG (2017) Urea hydrolysis in dairy cattle manure under different temperature, urea, and pH conditions. J Dairy Sci 100:2388–2394. 10.3168/jds.2016-11927 28088412

[CR40] Pedersen AR, Petersen SO, Schelde K (2010) A comprehensive approach to soil-atmosphere trace-gas flux estimation with static chambers. Eur J Soil Sci 61:888–902

[CR41] Punia H, Tokas J, Malik A, Satpal Rani A, Gupta P, Kumari A, Mor SM, Bhuker A, Kumar S (2020) Solar radiation and nitrogen use efficiency for sustainable agriculture. In: Resources use efficiency in agriculture, 177–212.

[CR42] Reynolds CM, Wolf DC, Armbruster JA (1985) Factors related to urea hydrolysis in soils. Soil Sci Soc Am J 49:104–108

[CR43] Rohe L, Well R, Lewicka-Szczebak D (2017) Use of oxygen isotopes to differentiate between nitrous oxide produced by fungi or bacteria during denitrification. Rapid Commun Mass Spectrom 31:1297–1312. 10.1002/rcm.7909 28556299

[CR44] Selbie DR, Cameron KC, Di HJ, Moir JL, Lanigan GJ, Richards KG (2014) The effect of urinary nitrogen loading rate and a nitrification inhibitor on nitrous oxide emissions from a temperate grassland soil. J Agric Sci 152:159–171. 10.1017/S0021859614000136

[CR45] Selbie DR, Buckthoght LE, Shepherd MA (2015) The challenge of the urine patch for managing nitrogen in grazed pasture systems. Adv Agron 129:229–292. 10.1016/bs.agron.2014.09.004

[CR46] Singh J, Saggar S, Bolan NS (2009) Influence of dicyandiamide on nitrogen transformation and losses in cow-urine-amended soil cores from grazed pasture. Anim Prod Sci 49:253–261. 10.1071/EA08200

[CR47] Somda ZC, Powell JM, Bationo A (1997) Soil pH and nitrogen following cattle and sheep urine deposition. Commun Soil Sci Plant Anal 28:1253–1268

[CR48] Sordi A, Dieckow J, Bayer C, Alburquerque MA, Piva JT, Zanatta JA, Tomazi M, da Rosa CM, de Moraes A (2014) Nitrous oxide emission factors for urine and dung patches in a subtropical Brazilian pastureland. Agric Ecosyst Environ 190:94–103

[CR49] Spek JW, Bannink A, Gort G, Hendriks WH, Dijkstra J (2012) Effect of sodium chloride intake on urine volume, urinary urea excretion, and milk urea concentration in lactating dairy cattle. J Dairy Sci 95:7288–7298 10.3168/jds.2012-5688 23063155

[CR50] Spek JW, Dijkstra J, van Duinkerken G, Hendriks WH, Bannink A (2013) Prediction of urinary nitrogen and urinary urea nitrogen excretion by lactating dairy cattle in northwestern Europe and North America: a meta-analysis. J Dairy Sci 96:4310–4322 10.3168/jds.2012-6265 23664347

[CR51] Spott O, Russow R, Stange CF (2011) Formation of hybrid N_2_O and hybrid N_2_ due to codenitrification: first review of a barely considered process of microbially mediated N-nitrosation. Soil Biol Biochem 43:1995–2011. 10.1016/j.soilbio.2011.06.014

[CR52] Steele KW, Judd MJ, Shannon PW (1984) Leaching of nitrate and other nutrients from a grazed pasture. N Z J Agric Res 27:5–11

[CR53] Stres B, Danevčič T, Pal L, Fuka MM, Resman L, Leskovec S, Hacin J, Stopar D, Mahne I, Mandic-Mulec I (2008) Influence of temperature and soil water content on bacterial, archaeal and denitrifying microbial communities in drained fen grassland soil microcosms. FEMS Microbiol Ecol 66:110–122 10.1111/j.1574-6941.2008.00555.x 18710395

[CR54] Syakila A, Kroeze C (2011) The global nitrous oxide budget revisited. Greenh Gas Meas Manag 1:17–26. 10.3763/ghgmm.2010.0007

[CR55] Talbot WD, Malcolm BJ, Cameron KC, Di HJ, Whitehead D (2021) Effect of timing of cattle urine deposition and pasture composition on nitrogen leaching losses. Soil Use Manag 37:723–735. 10.1111/sum.12652

[CR56] van der Weerden TJ, Clough TJ, Styles TM (2013) Using near-continuous measurements of N_2_O emission from urine-affected soil to guide manual gas sampling regimes. N Z J Agric Res 56:60–76

[CR57] van der Weerden TJ, Noble AN, Luo J, de Klein CAM, Saggar S, Giltrap D, Gibbs J, Rys G (2020) Meta-analysis of New Zealand’s nitrous oxide emission factors for ruminant excreta supports disaggregation based on excreta form, livestock type and slope class. Sci Total Environ 732:139235. 10.1016/j.scitotenv.2020.139235 32438176

[CR58] van Groenigen JW, Velthof GL, Van der Bolt FJE, Vos A, Kuikman PJ (2005) Seasonal variation in N_2_O emissions from urine patches: effects of urine concentration, soil compaction and dung. Plant Soil 273:15–27. 10.1007/s11104-004-6261-2

[CR59] Voglmeier K, Six J, Jocher M, Ammann C (2019) Grazing-related nitrous oxide emissions: from patch scale to field scale. Biogeosci 16:1685–1703

[CR60] Wang Y, Paul SM, Jocher M, Alewell C, Leifeld J (2022) Reduced nitrous oxide emissions from drained temperate agricultural peatland after coverage with mineral soil. Front Environ Sci 10:856599. 10.3389/fenvs.2022.856599 34426363

[CR61] Wang Y, Valach A, dos Reis Martins M, Ammann C (2025) Assessing nitrous oxide emissions from grass-clover ley within a crop rotation using measurements and modeling. Eur J Agron 170:127779

[CR62] Wei J, Ibraim E, Brüggemann N, Vereecken H, Mohn J (2019) First real-time isotopic characterisation of N_2_O from chemodenitrification. Geochim Cosmochim Acta 267:17–32. 10.1016/j.gca.2019.09.018

[CR63] Welten BG, Ledgard SF, Schipper LA, Waller JE, Kear MJ, Dexter MM (2013) Effects of prolonged oral administration of dicyandiamide to dairy heifers on excretion in urine and efficacy in soil. Agric Ecosyst Environ 173:28–36

[CR64] Whitehead DC, Raistrick N (1993) The volatilization of ammonia from cattle urine applied to soils as influenced by soil properties. Plant Soil 148:43–51

[CR65] Wrage-Mönnig N, Horn MA, Well R, Müller C, Velthof G, Oenema O (2018) The role of denitrification in the production of nitrous oxide revisited. Soil Biol Biochem 123:A3–A16. 10.1016/j.soilbio.2018.03.020

[CR66] Yu L, Harris E, Lewicka‐Szczebak D, Barthel M, Blomberg MR, Harris SJ, Johnson MS, Lehmann MF, Liisberg J, Müller C, Ostrom NE, Six J, Toyoda S, Yoshida N, Mohn J (2020) What can we learn from N2O isotope data? - Analytics, processes and modelling. Rapid Commun Mass Spectrom 34:e8858 10.1002/rcm.8858 32548934

